# 2011 space odyssey: spatialization as a mechanism to code order allows a close encounter between memory expertise and classic immediate memory studies

**DOI:** 10.3389/fpsyg.2014.00573

**Published:** 2014-06-10

**Authors:** Alessandro Guida, Magali Lavielle-Guida

**Affiliations:** ^1^Psychology Department, CRPCC, Université Rennes 2Rennes, France; ^2^Cabinet de Psychologie et d'OrthophonieSt Malo, France

**Keywords:** spatialization, immediate memory, expertise, long-term working memory, retrieval structures

In 2011 van Dijk and Fias with an innovative working memory paradigm showed for the first time that words to-be-remembered, presented sequentially at the center of a screen acquired a new spatial dimension: the first words of the sequence acquired a left spatial value while the last words acquired a right spatial value. In this article, we argue that this spatialization which putatively underpins how order is coded in immediate memory^1^ allows brid-ging the domain of memory expertise with classic immediate memory studies.

After briefly reviewing the mechanisms for coding order in immediate memory and the recent studies pointing toward spatialization as an explanatory mechanism, we will pinpoint similar mechanisms that are known to exist in memory expertise, particularly in the method of loci. We will terminate by analyzing what these similarities can tell us about expertise.

## How order is coded?

Surprisingly, this very fundamental question has not yet received a definitive answer. If one tries to naively think about a way order could be coded, generally the first idea that comes is chaining: items in a list to-be-remembered are just chained together by our cognitive system. And indeed, for more than four decades, this has been the most prominent idea among researchers (e.g., Wickelgren, 1965; Jordan, 1986; Lewandowsky and Murdock, 1989). This idea beyond being simple and intuitive, is also ancient since it roots back at least to Ebbinghaus (1885/2010). However, in the last two decades chaining models have lost ground, mostly because of experimental results. In immediate memory, error patterns (i.e., transposition and protrusion errors, Estes, 1991; Henson, 1996, 1999) and the distance effect (e.g., Hacker, 1980; Marshuetz et al., 2000) have been difficult to explain with the chaining concept.

## Positional tagging

Nowadays prominent models are of a positional kind (e.g., Anderson and Matessa, 1997; Burgess and Hitch, 1999; Brown et al., 2000, 2007; O'Reilly and Soto, 2001; Lewandowsky and Farrell, 2008a; Oberauer and Lewandowsky, 2011). Based on various studies (e.g., Dale, 1987; Poirier and Saint-Aubin, 1996; Mulligan, 1999; Engelkamp and Dehn, 2000; Henson et al., 2003), these models assume that item information and order information are coded and represented separately (for a review, see Marshuetz, 2005). Order is putatively coded through positional coding mechanisms, where a positional marker (or tag)–a context–is associated to each item. These contexts or positional markers can be temporal or not (Lewandowsky and Farrell, 2008b), but several studies seem to run against temporal markers (e.g., Lewandowsky and Brown, 2004, 2005; Lewandowsky et al., 2006), which favors non-temporal ones. Nonetheless if temporal tags are by definition well-known, the nature of non-temporal tags remains unknown (Lewandowsky and Farrell, 2008b)^2^. It could be an external context such as the environment or/and an internal context such as the inner states of the mind associated with each items.

## What does van dijck and fias (2011) study change concerning order coding?

In 2011 van Dijck and Fias proposed an alternative explanation of the SNARC (Spatial-Numerical Association of Response Codes) effect. This effect was first popularized by Dehaene et al. (1993). They used a classic parity judgment task where participants had to decide if a number was odd or even. However, the left-/right-hand key assignment was varied: the answer “even” (as the answer “odd”) was assigned for half of the trials to one hand and for the other half to the other hand. Results showed a SNARC effect, that is, small numbers triggered faster responses when participants answered with the left hand and large numbers triggered faster responses when participants answered with the right hand. According to Dehaene et al. (1993), the effect was due to the representation numbers have in (semantic) long-term memory (LTM), that of a mental line, which in western cultures increases from left to right (e.g., Dehaene et al., 1993; Göbel et al., 2011).

This LTM conception of the SNARC was disputed by van Dijck and Fias (2011) using a new paradigm. They proposed that the SNARC effect depended on the organization numbers assume in working memory. In the study, participants were presented five random numbers (ranging from 1 to 10) to-be-remembered in correct order. Numbers were displayed at the center of a screen. After the presentation phase, numbers ranging from 1 to 10 were displayed randomly at the center of screen. When a number to-be-remembered was displayed, participants had to execute a parity judgment task. As in Dehaene et al. (1993), the left-/right-hand key assignment was varied. But instead of the usual SNARC effect, results showed a Spatial-Positional Association of Response Codes (SPoARC) effect, that is, left hand responses were faster with numbers presented in the first positions of the to-be-remembered numbers (instead of small numbers in the SNARC effect) and right hand responses were faster with numbers presented in the last positions (instead of big numbers).

## A new positional tagging mechanism: spatialization

**Figure 1:**
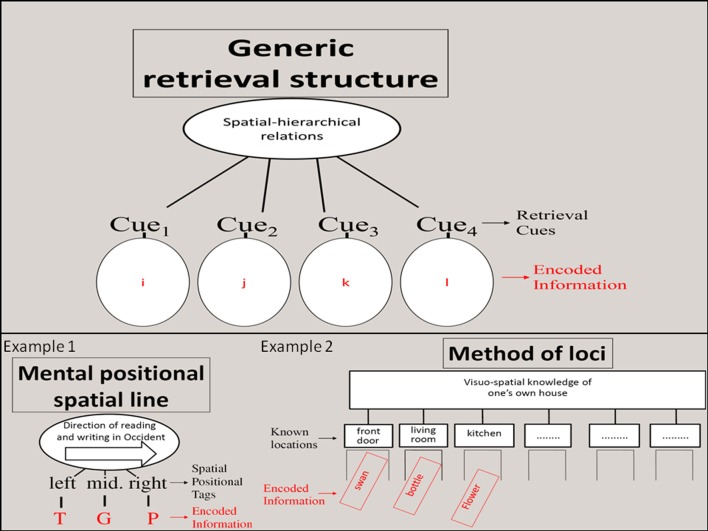
**Schematic representation of retrieval structures through two examples**. The upper part of the figure offers a generic and abstract representation of retrieval structures, from Ericsson and Kintsch (1995). The first example is taken from the spatial positional mental line and adapted from Guida (under review), it represent the encoding of three letters via three spatial positional tags. The second example is from the method of loci, and represents the encoding of three words via known locations used as retrieval cues.

This result and others (i.e., van Dijck et al., 2013; Guida, under review) suggest that the initial words of a sequence have a left spatial value while the last words of the same sequence have a right spatial value. Apparently individuals tend to create a spatial mental line based on the order items enter immediate memory (Example 1, Figure 1). This is highly compatible with the idea that in verbal immediate memory, items order is coded spatially, through spatialization. Given the fuzzy nature of non-temporal tags, this discovery could allow specifying the way items order is coded in immediate memory.

## What has spatialization got to do with memory expertise?

Since the very first (internal) mnemonic (Yates, 1966; Worthen and Hunt, 2011) which is thought to be the loci method proposed by Simonides of Ceos (556 BC–448 BC) and reported by Marcus Tullius Cicero in *De Oratore*, visuo-spatial processes have played a central role to enhance memory for verbal material. Concerning the loci method, Simonides of Ceos proposed to visualize a familiar route or a sequence of familiar locations (like rooms in one's own house) and use them to mentally store a list of words (Example 2, Figure 1), before a speech for example. Then during the speech, one would take a mental tour and retrieve each word via each familiar location (e.g., kitchen). Greek orators (Yates, 1966; Worthen and Hunt, 2011) soon became experts of the method of loci.

## The method of loci: expertise through spatialization

Of interest here, is the fact that the loci method necessitates to spatialize the items to-be-remembered in various locations. Moreover the method is not just an ancient oddity, the method efficiency has been confirmed since and still is nowadays. Memory experts (i.e., mnemonists) use it and several memory world records have been set with it. For example Pridmore (2013) was the first man to break the 30-s barrier in the Speed Cards discipline, which necessitates to memorize the order of a shuffled deck of cards. To do so, he used a system based on the method of loci, he spatialized groups of two cards the long of a familiar route.

## Is there a theory of expertise that supports the loci method phenomenology which points toward spatialization?

Even if mnemonics and memory expertise are very ancient (certainly due to oral tradition, see Rubin, 1997; Ong, 2012), grounded cognitive theories describing them are recent. It could be argued that the first complete theoretical contribution on mnemonic expertise (but see the Chunking theory, Chase and Simon, 1973) was Chase and Ericsson's (1981) Skilled memory theory, which was to be completed with the Long-term working memory (LT-WM) theory (Ericsson and Kintsch, 1995).

## Long-term working memory and retrieval structures

In order to explain memory expertise, Chase and Ericsson (1981) proposed three principles: the significant encoding, the structured retrieval and the principle of acceleration. The first principle proposes that in order to swiftly and reliably store items in LTM, information need to be transformed into meaningful units. Of interest here is the second principle which states that to increase mnemonic performances, hierarchical spatial cognitive structures, named retrieval structures (for a discussion, see Ericsson and Kintsch, 2000; Gobet, 2000a,b) can be used to encode and retrieve items from LTM. These structures constitute an internal artificial context to which items are linked to. In the loci method, it is done via the visuo-spatial knowledge of a sequence of familiar locations. Each location is a retrieval cue, and all the cues together constitute a retrieval structure (Figure 1). The skilled memory theory was first proposed to account for the performances of experts capable to increase their digit span above 80. The LT-WM (Ericsson and Kintsch, 1995) was a generalization of this theory to all activities and to all individuals, experts and novices.

## What does spatialization as a link between classic immediate memory studies and memory expertise bring as psychological perspectives on expertise?

Notwithstanding Ericsson and Kintsch's (1995) generalization, the LT-WM theory remains underused in the classic domain of verbal immediate memory (but, see Guida et al., 2009, 2013). As stated by Ericsson and Kintsch (1995, p. 217) concerning the Skilled memory theory (but the same can be said for LT-WM), even if this theoretical construct is largely accepted as accounting for experts, “several investigators (e.g., Schneider and Detweiler, 1987; Carpenter and Just, 1989; Baddeley, 1990) have voiced doubts about its generalizability.” Retrieval structures are often dismissed because considered too artificial or idiosyncrasies to be reserved to experts. Thank to van Dijck and Fias's (2011) study, this could change.

## Retrieval structure as spatialization: a genuine and universal process

As seen previously, van Dijck and colleagues' results (van Dijck and Fias, 2011; van Dijck et al., 2013; see also Guida, under review) clearly point toward the idea that in all-comers, spatial processes are also at stake in verbal immediate memory. When comparing retrieval structures such as in the method of loci and spatial positional tags, the similarities are striking (Figure 1). In both cases, a virtual spatial construct, used as a context, is associated to the incoming information. And the context can later be used to retrieve the items. Even if the mental line (Dehaene et al., 1993; van Dijck and Fias, 2011) used by all-comers is much simpler and lesser sophisticated, compared to mnemonists using the method of loci, spatialization seems the same underpinning process. If this standpoint is adopted, then it becomes more explicit why the loci method is so ancient and efficient: because experts' spatialization via retrieval structures roots on basic processes that all individuals can use. *Ipso facto*, retrieval structures stop being idiosyncrasies to be reserved to experts.

The link between both kinds of spatialization becomes even more tangible when considering that the spatial mental line could also be due to our expertise, in this case in mastering the writing system. In fact the orientation and direction of our mental line varies according to reading/writing habits (e.g., Dehaene et al., 1993; Shaki et al., 2009; Göbel et al., 2011; for the influence of reading habits on visuo-spatial processes, e.g., see Maass and Russo, 2003; Dobel et al., 2007). Therefore, it is very plausible that our reading and writing habits foster our spatial mental line.

When considering the privileged link between space and memory, it is also interesting to conclude taking a brief glance to anthropology, which shows that this link seems to be far more ancient than our reading habits and already present in non-literate societies. In fact myths around the world have often been linked to specific locations. This “myth spatialization” can be found in the Tobriand culture from Papua New Guinea for example, or in the Australian aborigines famous songlines (Chatwin, 1987) or even in Zunis' legends from southwestern United States. In all these cases, “spatial location functions as a mnemonic device for the recall of a corpus of myth” (Harwood, 1976, p. 783). Building on Harwood's (1976) myth spatialization, the loci method can be considered as a phylogenetic protraction of the myth spatialization, and the mental spatial line as an ontogenetic protraction of our reading habits.

### Conflict of interest statement

The authors declare that the research was conducted in the absence of any commercial or financial relationships that could be construed as a potential conflict of interest.

## Footnotes

^1^Immediate memory is an umbrella term for working memory and short-term memory.

^2^Lewandowsky and Farrell (2008b) wrote: “The use of context markers does, however, entail a cost: As in many other models (e.g., SEM; Henson, 1998), the structure of the markers across positions is assumed rather than explained by the model. That is, although it is entirely plausible to postulate that the contexts of adjacent items are more similar to each other than the contexts of items separated by intervening events, the precise form of their similarity relationship is not derived from the model's architecture. Are there any candidate mechanisms on the horizon that might permit a more principled derivation of context markers?.”

## References

[B1] Anderson J. R. Matessa M. (1997). A production system theory of serial memory. Psychol. Rev. 104, 728–748 10.1037/0033-295X.104.4.728

[B3] Baddeley A. D. (1990). Human Memory: Theory and Practice. Boston, MA: Allyn & Bacon

[B4] Brown G. D. A. Neath I. Chater N. (2007). A temporal ratio model of memory. Psychol. Rev. 114, 539–576 10.1037/0033-295X.114.3.539 17638496

[B5] Brown G. D. A. Preece T. Hulme C. (2000).Oscillator-based memory for serial order. Psychol. Rev. 107, 127–181 10.1037/0033-295X.107.1.127 10687405

[B6] Burgess N. Hitch G. J. (1999). Memory for serial order a network model of the phonological loop and its timing. Psychol. Rev. 106, 551–581 10.1037/0033-295X.106.3.551

[B7] Carpenter P. A. Just M. A. (1989). The role of working memory in language comprehension, in Complex Information Processing: The Impact of Herbert A. Simon, eds Klahr D. Kotovsky K. (Hillsdale, NJ: Erlbaum), 31–68

[B8] Chase W. G. Ericsson K. A. (1981). Skilled memory, in Cognitive Skills and their Acquisition, ed Anderson J. R. (Hillsdale, NJ: Lawrence Erlbaum Associates), 141–189

[B9] Chase W. G. Simon H. A. (1973). Perception in chess. Cogn. Psychol. 4, 55–81 10.1016/0010-0285(73)90004-2

[B10] Chatwin B. (1987) The Songlines. London: Jonathan Cape Ltd.

[B11] Dale R. H. (1987). Similarities between human and animal spatial memory: item and order information. Anim. Learn. Behav. 15, 293–300 10.3758/BF03205022

[B12] Dehaene S. Bossini S. Giraux P. (1993). The mental representation of parity and numerical magnitude. J. Exp. Psychol. Gen. 122, 371–396 10.1037/0096-3445.122.3.371

[B13] Dobel C. Diesendruck G. Bolte J. (2007). How writing system and age influence spatial representations of actions: a developmental, cross-linguistic study. Psychol. Sci. 18, 487–491 10.1111/j.1467-9280.2007.01926.x 17576259

[B14] Ebbinghaus H. (1885/2010). La Mémoire: Recherches de Psychologie Expérimentale. Paris: L'Harmattan

[B16] Engelkamp J. Dehn D. M. (2000). Item and order information in subject-performed tasks and experimenter-performed tasks. J. Exp. Psychol. Learn. Mem. Cogn. 26, 671–682 10.1037/0278-7393.26.3.671 10855425

[B17] Ericsson K. A. Kintsch W. (1995). Long-term working memory. Psychol. Rev. 102, 211–245 10.1037/0033-295X.102.2.211 7740089

[B18] Ericsson K. A. Kintsch W. (2000). Shortcomings of generic retrieval structures with slots of the type of Gobet (1993) proposed and modeled. Br. J. Psychol. 91, 571–590 10.1348/000712600161998 11104179

[B19] Estes W. K. (1991). On types of item coding and sources of recall in short-term memory, in Relating Theory and Data: In Honor of Bennet B. Murdock, eds Hockley W. E. Lewandowsky S. (Hillsdale, NJ: Lawrence Erlbaum Associates Inc.), 175–194

[B20] Göbel S. M. Shaki S. Fischer M. H. (2011). The cultural number line: a review of cultural and linguistic influences on the development of number processing. J. Cross Cult. Psychol. 42, 543–565 10.1177/0022022111406251

[B21] Gobet F. (2000a). Some shortcomings of long-term working memory. Br. J. Psychol. 91, 551–570 10.1348/000712600161989 11104178

[B22] Gobet F. (2000b). Retrieval structures and schemata: a brief reply to Ericsson and Kintsch. Br. J. Psychol. 91, 591–594 10.1348/000712600162005 11104179

[B24] Guida A. Gras D. Noel Y. Le Bohec O. Quaireau C. Nicolas S. (2013). The effect of long-term working memory through personalization applied to free recall: uncurbing the primacy effect enthusiasm. Mem. Cognit. 41, 571–587 10.3758/s13421-012-0284-3 23297048

[B25] Guida A. Tardieu H. Nicolas S. (2009). The personalisation method applied to a working memory task: evidence of long-term working memory effects. Eur. J. Cogn. Psychol. 21, 862–896 10.1080/09541440802236369

[B27] Hacker M. J. (1980). Speed and accuracy of recency judgments for events in short-term memory. J. Exp. Psychol. Hum. Learn. 6, 651–675 10.1037/0278-7393.6.6.651

[B28] Harwood F. (1976). Myth, memory, and the oral tradition: Cicero in the Trobriands. Am. Anthropol. 78, 783–796 10.1525/aa.1976.78.4.02a00040

[B29] Henson R. N. A. (1996). Short-Term Memory for Serial Order. Unpublished doctoral dissertation, University of Cambridge, Cambridge.

[B30] Henson R. N. A. (1998). Short-term memory for serial order: the start-end model. Cogn. Psychol. 36, 73–137 10.1006/cogp.1998.0685 9721198

[B31] Henson R. N. A. (1999). Coding position in short-term memory. Int. J. Psychol. 34, 403–409 10.1080/002075999399756

[B31a] Henson R. N. A. Hartley T. Burgess N. Hitch G. Flude B. (2003). Selective interference with verbal short-term memory for serial order information: a new paradigm and tests of a timing signal hypothesis. Q. J. Exp. Psychol. A 56, 1307–1334 10.1080/02724980244000747 14578087

[B32] Jordan M. I. (1986). Serial Order: a Parallel Distributed Approach (ICS Report 8604). San Diego: University of California, Institute for Cognitive Science

[B33] Lewandowsky S. Brown G. D. A. (2004). Time does not cause forgetting in short-term serial recall. Psychon. Bull. Rev. 11, 771–790 10.3758/BF03196705 15732687

[B34] Lewandowsky S. Brown G. D. A. (2005). Serial recall and presentation schedule: a micro-analysis of local distinctiveness. Memory 13, 283–292 10.1080/09658210344000251 15948613

[B35] Lewandowsky S. Brown G. D. A. Wright T. Nimmo L. M. (2006). Timeless memory: Evidence against temporal distinctiveness models of short-term memory for serial order. J. Mem. Lang. 54, 20–38 10.1016/j.jml.2005.08.004

[B36] Lewandowsky S. Farrell S. (2008a). Phonological similarity in serial recall: constraints on theories of memory. J. Mem. Lang. 58, 429–448 10.1016/j.jml.2007.01.005

[B37] Lewandowsky S. Farrell S. (2008b). Short-term memory: new data and a model, in The Psychology of Learning and Motivation, Vol. 49, ed Ross B. H. (London: Elsevier), 1–48

[B38] Lewandowsky S. Murdock B. B. Jr. (1989). Memory for serial order. Psychol. Rev. 96, 25–57 10.1037/0033-295X.96.1.25

[B39] Maass A. Russo A. (2003). Directional bias in the mental representation of spatial events: nature or culture? Psychol. Sci. 14, 296–301 10.1111/1467-9280.14421 12807400

[B42] Marshuetz C. (2005). Order information in working memory: an integrative review of evidence from brain and behavior. Psychol. Bull. 131, 323–339 10.1037/0033-2909.131.3.323 15869331

[B43] Marshuetz C. Smith E. E. Jonides J. DeGutis J. Chenevert T. L. (2000). Order information in working memory: fMRI evidence for parietal and prefrontal mechanisms. J. Cogn. Neurosci. 12, 130–144 10.1162/08989290051137459 11506653

[B46] Mulligan N. W. (1999). The effects of perceptual interference at encoding on organization and order: Investigating the roles of item-specific and relational information. J. Exp. Psychol. Learn. Mem. Cogn. 25, 54–69 10.1037/0278-7393.25.1.54 9949708

[B47] Oberauer K. Lewandowsky S. (2011). Modeling working memory: a computational implementation of the time-based resource-sharing theory. Psychon. Bull. Rev. 18, 10–45 10.3758/s13423-010-0020-6 21327362

[B48] Ong W. J. (2012). Orality and Literacy: The Technologizing of the Word. London: Routledge

[B49] O'Reilly R. C. Soto R. (2001). A model of the phonological loop: generalization and binding, in Advances in Neural Information Processing Systems, eds Dietterich T. G. Becker S. Ghahramani Z. (Cambridge, MA: MIT Press), 83–90

[B50] Poirier M. Saint-Aubin J. (1996). Immediate serial recall, word frequency, item identity and item position. Can. J. Exp. Psychol. 50, 408–412 10.1037/1196-1961.50.4.408 9025332

[B51] Pridmore B. (2013). How to be Clever. Available online at: http://www.lulu.com/

[B54] Rubin D. C. (1997). Memory in Oral Traditions: The Cognitive Psychology of Epic, Ballads, and Counting-out Rhymes. New York, NY: Oxford University Press

[B55] Schneider W. Detweiler M. (1987). A connectionist/control architecture for working memory, in The Psychology of Learning and Motivation, ed Bower G. H. (New York, NY: Academie Press), 54–119

[B56] Shaki S. Fischer M. H. Petrusic W. M. (2009). Reading habits for both words and numbers contribute to the SNARC effect. Psychon. Bull. Rev. 16, 328–331 10.3758/PBR.16.2.328 19293102

[B57] van Dijck J.-P. Abrahamse E. L. Majerus S. Fias W. (2013). Spatial attention interacts with serial order retrieval in verbal working memory. Psychol. Sci. 24, 1854–1859 10.1177/0956797613479610 23863755

[B58] van Dijck J. P. Fias W. (2011). A working memory account for spatial-numerical associations. Cognition 119, 114–119 10.1016/j.cognition.2010.12.013 21262509

[B60] Wickelgren W. A. (1965). Short-term memory for phonemically similar lists. Am. J. Psychol. 78, 567–574 10.2307/1420917 5839924

[B61] Worthen J. B. Hunt R. R. (2011). Mnemonology: Mnemonics for the 21st Century. Hove: Psychology Press

[B62] Yates F. A. (1966). The Art of Memory. Chicago: University of Chicago Press

